# A New Algorithm for Cancer Biomarker Gene Detection Using Harris Hawks Optimization

**DOI:** 10.3390/s22197273

**Published:** 2022-09-26

**Authors:** Halah AlMazrua, Hala AlShamlan

**Affiliations:** Information Technology Department, College of Computer and Information Sciences, King Saud University (KSU), Riyadh 11451, Saudi Arabia

**Keywords:** bio-inspired algorithms, bioinformatics, cancer classification, evolutionary algorithm, feature selection, gene expression, Harris Hawks Optimization, *k*-nearest neighbor, support vector machine

## Abstract

This paper presents two novel swarm intelligence algorithms for gene selection, HHO-SVM and HHO-KNN. Both of these algorithms are based on Harris Hawks Optimization (HHO), one in conjunction with support vector machines (SVM) and the other in conjunction with *k*-nearest neighbors (*k*-NN). In both algorithms, the goal is to determine a small gene subset that can be used to classify samples with a high degree of accuracy. The proposed algorithms are divided into two phases. To obtain an accurate gene set and to deal with the challenge of high-dimensional data, the redundancy analysis and relevance calculation are conducted in the first phase. To solve the gene selection problem, the second phase applies SVM and *k*-NN with leave-one-out cross-validation. A performance evaluation was performed on six microarray data sets using the two proposed algorithms. A comparison of the two proposed algorithms with several known algorithms indicates that both of them perform quite well in terms of classification accuracy and the number of selected genes.

## 1. Introduction

Approximately 10 million people worldwide die from cancer every year, or one in every six deaths, according to the WHO [1]. Early diagnosis and treatment can reduce the cancer mortality rate. Wrong classifications and predictions cause serious harm to patients and their families [2]. Generally, microarray data are employed in cancer research, where early detection of cancer can greatly influence the treatment and survival rate [3]. Nevertheless, microarray data suffer from high dimensionality issues since the number of genes far outnumbers the number of samples, with the result of the so-called “curse of dimensionality”. When the dimensionality of a data set rises significantly, it can be difficult to demonstrate the statistical significance of the results [4].

There have been four approaches to solving the “curse of dimensionality”: filtering, wrapper, embedded, and hybrid methods [5]. The filtering method evaluates the relevance of features as scores based only on property values. It is possible to sort features by their scores and to remove low-scoring features. In wrapper methods, the analysis model is embedded within the search for appropriate features. Embedded methods search for an optimal subset of features as part of the analysis algorithm. Hybrid methods combine two methods for selecting features to take advantage of both [6].

Two feature-selection methods based on wrapper-based algorithms are presented in this paper, both of which employ the Harris Hawks Optimizer (HHO) to select the most informative genes for classification and achieve high accuracy: HHO-SVM works in conjunction with support vector machines (SVM), and HHO-KNN works in conjunction with the *k*-nearest neighbors (*k*-NN) algorithm. To evaluate the effectiveness of both HHO-SVM and HHO-KNN, we compared the results from six microarray cancer data sets to several recently published techniques. In both binary and multiclass classifications, HHO-KNN and HHO-SVM appear to be able to achieve higher classification accuracy with a smaller number of genes selected. This paper aim test HHO as a feature selection method and view its effectiveness. This paper will answer those two questions: Can we use HHO as a feature selection method on well-known cancer gene microarray datasets? In addition, which classifier works best with HHO?

The paper is structured as follows: Section 2 describes how HHO was inspired and the mathematical modeling that went into it. In Section 3, we introduce our proposed HHO-SVM and HHO-KNN approaches to gene selection. Discussions and experimental results are presented in Section 4. Finally, the conclusion is given in Section 5.

## 2. The Harris Hawks Optimizer

### 2.1. Inspiration

The Harris Hawks Optimizer (HHO) is a swarm computation method that was developed by Heidari et al. in 2019 [7]. This algorithm was inspired by the cooperative hunting and chasing behavior exhibited by Harris’s hawks, particularly “surprise pounces” or “the seven kills.” In a cooperative attack, numerous hawks coordinate their efforts and simultaneously attack a rabbit that has shown itself.

The attack could well be accomplished quickly by catching the surprised prey in a matter of seconds; however, depending on the prey’s actions and ability to flee, the attack may include repeated, short, fast dives near the prey over the course of many minutes. According to the changing circumstances and the prey’s escape patterns, Harris’s hawks can demonstrate a variety of chasing styles. Generally, tactics are changed when the party’s strongest hawk (leader) goes after the prey but loses it, at which point another party member continues the chase. It is common to observe these switches in a variety of settings because they are used to confuse escaping rabbits. Moreover, the rabbit has no way of regaining its defensive abilities when a new hawk begins to chase it, and it is unable to escape the attacking team since any hawk, usually the most experienced and powerful, captures the exhausted rabbit and shares it with the rest of the team.

### 2.2. Mathematical Modeling

Hawks are known to chase their prey by tracing, encircling, and eventually striking and killing. The mathematical model, which is based on hawks’ hunting behaviors, comprises three various stages: exploration, transition between exploration and exploitation, and exploitation. At each stage of the hunt, the Harris’s hawks are the candidate solutions, and the targeted prey is the best candidate solution (almost the optimal).

As they search for prey, Harris’s hawks use two different exploration techniques. Candidate solutions are designed to be as close to the prey as possible, while the best is the one that is the intended prey. First, Harris’s hawks choose a spot by considering the locations of other hawks and their prey. In the second method, the hawks wait on random tall trees. Using Equation (1), the two methods can be simulated with equal odds of *q*:

(1) $$x\left(t+1\right)=\left\{\begin{array}{cc} x_{random}\left(t\right)-r_{1}\left|x_{random}\left(t\right)-2r_{2}x\left(t\right)\right| & q⩾0.5\\ x_{rabbit}\left(t\right)-x_{mean}\left(t\right)-r_{3}\left(LB+r_{4}\left(UB-LB\right)\right) & q<0.5 \end{array}\right.$$

- Vector x(t) is the current hawk position, whereas vector x(t+1) is the hawk’s position at the next iteration.
- The hawk $x_{random}\left(t\right)$ is selected at random from the population.
- The rabbit position is $x_{rabbit}\left(t\right)$.
- $q,r_{1},r_{2},r_{3}$ and $r_{4}$ are randomly generated numbers inside (0,1).
- LB and UB are the upper and lower bounds of variables.
- $x_{mean}\left(t\right)$ is the average position of the current population of hawks, which is calculated as shown in Equation (2).

(2) $$x_{mean}\left(t\right)=\frac{1}{N}\sum_{i=1}^{N}x_{i}\left(t\right)$$

- *t* is the total number of iterations.
- $x_{i}\left(t\right)$ is the position for each hawk in iteration *t*.
- The total number of hawks is represented by *N*.

The algorithm switches from exploration to exploitation (transition from exploration to exploitation) depending on the rabbit’s running or escaping energy, as shown in Equation (3).

(3) $$E=2E_{0}\left(1-\frac{t}{Max\_iter}\right)$$

- *E* represents the prey’s escaping energy.
- The initial state of the energy is indicated by $E_{0}$, which changes randomly inside (−1, 1) at each iteration.

When |E|⩾1, hawks seek out more areas to investigate the rabbit’s whereabouts; alternatively, the exploitation stage begins. The algorithm formulates the rabbit’s escape success p⩾0.5 or failure p<0.5 with an equal chance *p*. The Hawks also will also carry out a soft |E|⩾0.5 or hard siege |E|<0.5, based on the rabbit’s energy. The soft siege is defined as in Equations (4)–(6).

(4) $$x\left(t+1\right)=\Delta x\left(t\right)-E|J\cdot x_{rabbit}\left(t\right)-x\left(t\right)$$

(5) $$\Delta x\left(t\right)=x_{rabbit}\left(t\right)-x\left(t\right)$$

(6) J=2(1−random)

- The difference between the hawk and rabbit positions is represented by Δx(t).
- *J* is a random number used to generate the rabbit’s random jump force.

A hard siege, on the other hand, can be calculated as follows in Equation (7):

(7) x(t+1)=x(t)−E|Δx(t)|

A soft siege with repeated fast dives is attempted when |E|⩾0.5 and p<0.5, as the rabbit could successfully escape. The hawks have the option of selecting the best dive. Lévy flight is employed to imitate the prey’s hopping. The hawks’ next action is calculated as shown in Equation (8) to determine whether the dive is successful or not.

(8) $$k=x_{rabbit}\left(t\right)-E\left|J\cdot x_{rabbit}\left(t\right)-x\left(t\right)\right|$$

The hawks will dive following Equation (9), the Lévy flight *L* pattern, if the previous dive turns out to be ineffective.

(9) z=k+RandomVector·L(dim)

- The problem dimension dim is the size of the random vector RandomVector, and dim is the dimension of the problem.

Equation (10) has been used to update the final soft-siege rapid dives

(10) $$x\left(t+1\right)=\left\{\begin{array}{ccc} k & if & f(k)<f(x(t))\\ z & if & f(z)<f(x(t)) \end{array}\right.$$

Equations (8) and (9) are used to calculate *k* and *z*, respectively. A hard siege with progressive rapid dives occurs when |E|⩾0.5 and p<0.5 are not sufficient for the rabbit to flee, as it no longer possesses enough energy. The rabbit’s *z* is calculated via Equation (9), while *k* is updated using Equation (11).

(11) $$k=x_{rabbit}\left(t\right)-E\left|J\cdot x_{rabbit}\left(t\right)-x_{mean}\left(t\right)\right|$$

## 3. Proposed Algorithm

In this section, how the two proposed algorithms work will be described in detail. We combined HHO with SVM and *k*-NN to develop two approaches, HHO-SVM and HHO-KNN, for solving the microarray high dimensionality issue to find the most meaningful genes and compare between SVM and *k*-NN classifiers to find which one gives the best accuracy and selects the fewest genes. The fitness function that is used is the error rate.

**Figure 1:**
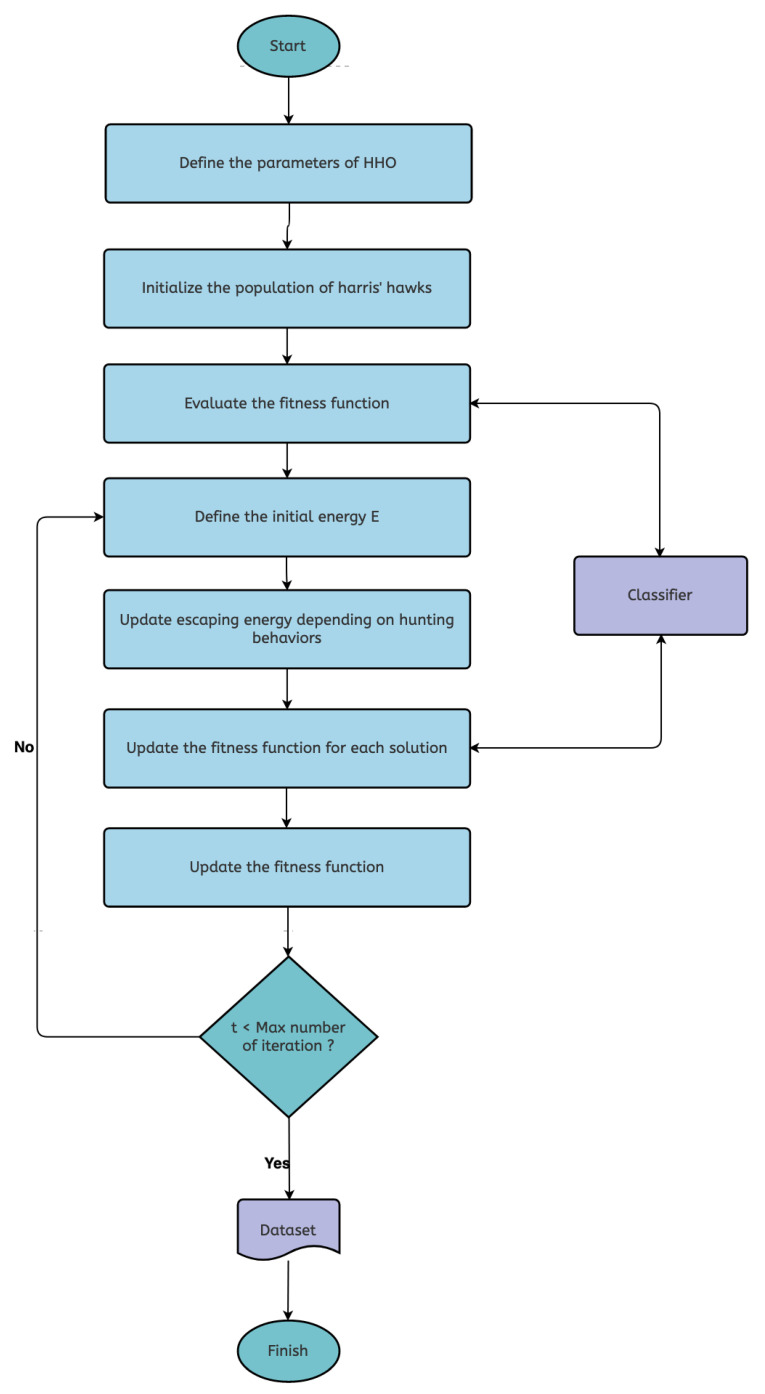
HHO-SVM and HHO-KNN flowchart.

The steps of both the HHO-SVM and HHO-KNN algorithms are shown in Figure 1. In addition, in Algorithm 1, we present pseudo code for the HHO algorithm.

To evaluate the performance of the two proposed approaches, leave-one-out cross-validation (LOOCV) was used to avoid model overfitting of both classifiers to calculate the accuracy. All samples except one are used as testing data in LOOCV, with the remaining sample used as training data. This is repeated until all samples have been tested. Based on *N* times of classification, the LOOCV calculates the average accuracy.
**Algorithm 1:** Pseudo-Code of HHO Algorithm.
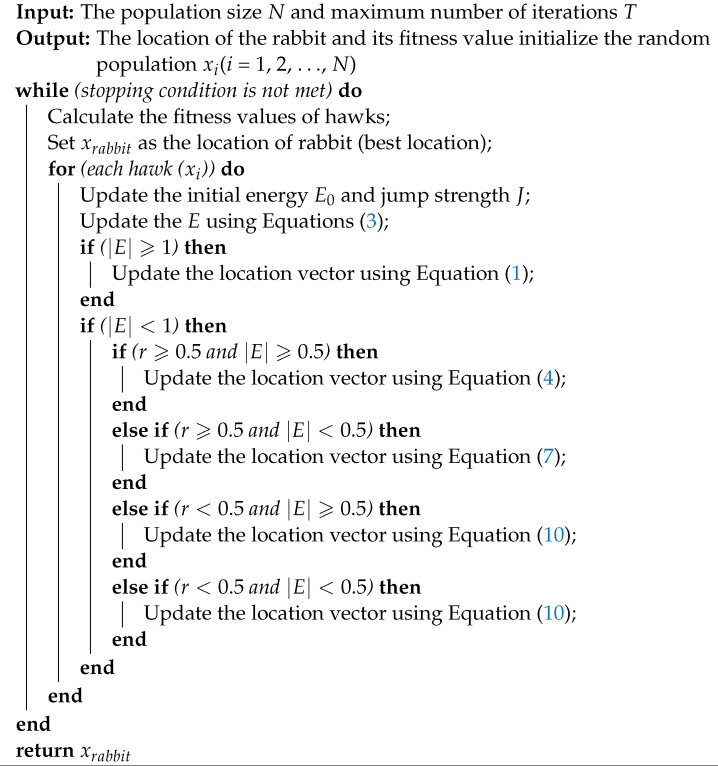


## 4. Experimental Results and Discussions

The exploratory approach, the findings of implementing the proposed algorithms to microarray cancer data sets, and the gene expression data sets used in the study are all described in this section.

### 4.1. Data Sets

In our study, we used two publicly available microarray cancer data sets and binary and multiclass data sets. The performance and effectiveness of the two algorithms were evaluated by evaluating six benchmark microarray data sets. The three binary data sets used were for colon tumors [8], lung cancer [9], and leukemia3 [10]. In addition, there were three multiclass data sets, which were leukemia2 [8], lymphoma [11], and SRBCT [11]. A detailed breakdown of the experimental data sets on the basis of diverse samples and classes can be found in Table 1.

### 4.2. Parameter Settings

To determine the most suitable solution, SVM and *k*-NN classifiers were used. Since k=7 performed well across all test sets, it was used in the experiments. There are two significant factors that influence the practicality of a method: its iterations (Max_iter) and its dimensions. In addition to *k*, dimensions, UB, and LB, there are other parameters, which can be found in Table 2.

### 4.3. Results and Analysis

Features are selected to improve the accuracy of the classification while lowering the number of features being used. Each data set was processed with the two algorithms on a different number of features. We applied the proposed techniques in each cancer data set by using 1 to 30 genes. For evaluating the experimental results, both HHO-KNN and HHO-SVM were applied to binary and multiclass high-dimensional microarray cancer data sets for selecting genes. There were two metrics used in our comparison: classification accuracy and the number of genes selected for cancer classification. Here are the experimental results for all of the cancer data sets that were used.

On the colon data set, Table 3 shows the best, worst, and average classification accuracy using the HHO-KNN and HHO-SVM algorithms. Interestingly, the highest classification accuracy obtained was the same when either the *k*-NN or the SVM classifier was applied with 90.32%. However, with SVM, the number of selected genes was 10 genes that is lower than the selected genes for *k*-NN, with 16 genes.

Looking at Table 4 for Leukemia2 data set results, we can see both HHO-KNN and HHO-SVM by selecting the same number of genes (11 genes); the SVM classifier is more accurate, 97.22%.

The results of implementing HHO-SVM and HHO-KNN algorithms in the leukemia3 data set are shown in Table 5. When *k*-NN was used, the best classification accuracy was achieved when 25 genes were selected. The classification accuracy increased to 90.28% for *k*-NN and 84.72% for SVM.

The accuracy performance of best, average, and worst HHO-SVM and HHO-KNN algorithms in the Lung data set is presented in Table 6. It shows the highest accuracy of 100% when 2 or 10 genes are selected for both the *k*-NN and SVM classifiers.

Table 7 shows the best, worst, and average classification accuracy of Lymphoma data set for applying the HHO-KNN and HHO-SVM algorithms. It achieve an accuracy of 100% in most cases for both classifiers, but the selected genes for *k*-NN was lower than SVM to achieve 100% accuracy with 2 genes in *k*-NN and 3 genes for SVM.

Table 8 compares the average, best, and worst accuracy performance on the implementation of HHO-SVM and HHO-KNN algorithms in the SRBCT data set. The highest accuracy was when 29 genes are selected with 92.77% for SVM and 91.57% for *k*-NN.

### 4.4. Comparative Evaluations

Comparing and evaluating the performance of HHO-SVM and HHO-KNN against the other bio-inspired metaheuristic gene selection algorithms was an important part of our evaluation. Table 9 shows how our findings compare based on accuracy and the number of genes selected.

As can be seen in Table 9 for lung and lymphoma, the HHO-KNN accuracy outperformed the other bio-inspired gene selection algorithms since it reached 100% classification accuracy, and the number of selected genes is smaller than the other methods. HHO-SVM for the lung data set outperformed the other bio-inspired gene selection algorithms. In addition, as can be seen from the table, HHO-KNN and HHO-SVM performed better than their competitor (BQPSO) on the colon data set.

## 5. Conclusions

Our study proposes two new feature selection techniques using Harris Hawks Optimization (HHO) combined with support vector machines (SVM) and the *k*-nearest neighbors (*k*-NN) algorithm for high-dimensional cancer gene selection and classification. The objective of this study was to devise a new algorithm for solving gene selection problems based on bio-inspired principles. Using HHO-SVM and HHO-KNN on six binary and multiclass high-dimensional cancer microarray data sets, we have shown that in terms of classification accuracy and the number of chosen genes, our two algorithms are better than several other algorithms in finding useful informative genes.

The experimental findings are displayed by using gene expression data sets, and with all of the data sets, we observe that we only achieved 100% accuracy for the lung and lymphoma datasets. Additionally, the accuracy obtained for the entire dataset with the KNN classifier and the SVM classifier is greater than 90%. Last but not least, on all datasets except the Leukemia3 dataset, HHO-KNN outperformed HHO-SVM. As well as discovering the tremendous promise for HHO when used alone, we recommend combining HHO with another wrapper bio-inspired feature selection methodology to produce a hybrid method that enhances HHO accuracy for future works while picking fewer genes.

## Figures and Tables

**Table 1:** Description of microarray data sets.

Data Set	No. Total Genes	No. Samples	No. Classes
Colon Tumor [8]	2000	62	2
Lung Cancer [9]	7129	96	2
Leukemia2 [8]	7129	72	3
Leukemia3 [10]	7129	72	2
SRBCT [11]	2308	83	4
Lymphoma [11]	4026	66	3

**Table 2:** Parameter settings for HHO-SVM and HHO-KNN.

Parameter	Value
Dimension	No. genes in data set
No. iterations (Max_iter)	100
Lower bound (LB)	0
Upper bound (UB)	1
No. Harris’s hawks (SearchAgents_no)	10
No. runs (m)	30
*k*	7

**Table 3:** Colon data set results.

	No. Genes	Best	Average	Worst
HHO-KNN	20	82.26%	75.92%	64.52%
	**16**	**90.32%**	74.42%	53.23%
	10	88.71%	74.65%	61.29%
	5	83.87%	68.12%	53.23%
	2	79.03%	64.01%	48.39%
HHO-SVM	20	85.48%	76.21%	62.90%
	16	87.10%	74.48%	56.45%
	**10**	**90.32%**	73.94%	51.61%
	5	83.87%	69.02%	56.45%
	2	74.19%	64.16%	51.61%

**Table 4:** Leukemia2 data set results.

	No. Genes	Best	Average	Worst
HHO-KNN	16	66.67%	61.25%	54.17%
	**11**	**94.44%**	64.29%	52.78%
	6	73.61%	64.12%	55.56%
	2	72.22%	61.62%	48.61%
HHO-SVM	16	68.06%	64.95%	62.50%
	**11**	**97.22%**	66.17%	58.33%
	6	72.22%	65.09%	58.33%
	2	72.22%	65.32%	59.72%

**Table 5:** Leukemia3 data set results.

	No. Genes	Best	Average	Worst
HHO-KNN	30	69.44%	59.07%	50.00%
	**25**	**90.28%**	58.52%	45.83%
	20	63.89%	55.42%	44.44%
	15	59.72%	52.69%	43.06%
	5	61.11%	52.82%	43.06%
HHO-SVM	30	69.44%	55.97%	37.50%
	**25**	**84.72%**	58.19%	50.00%
	20	65.28%	53.43%	38.89%
	15	63.89%	53.33%	38.89%
	5	66.67%	55.00%	40.28%

**Table 6:** Lung data set results.

	No. Genes	Best	Average	Worst
HHO-KNN	19	98.96%	92.38%	83.33%
	10	100.00%	93.70%	86.46%
	**2**	**100.00%**	91.57%	84.38%
	1	97.92%	89.65%	85.42%
HHO-SVM	19	95.83%	90.25%	89.58%
	10	100.00%	90.90%	87.50%
	**2**	**100.00%**	92.09%	86.46%
	1	97.92%	89.76%	86.46%

**Table 7:** Lymphoma data set results.

	No. Genes	Best	Average	Worst
HHO-KNN	12	98.48%	92.31%	80.30%
	10	100.00%	92.22%	77.27%
	3	100.00%	77.23%	60.61%
	**2**	**100.00%**	73.63%	60.61%
	1	75.76%	66.36%	56.06%
HHO-SVM	12	100.00%	93.43%	80.30%
	10	100.00%	92.66%	72.73%
	**3**	**100.00%**	78.35%	65.15%
	2	96.97%	74.01%	65.15%
	1	81.82%	70.61%	66.67%

**Table 8:** SRBCT data set results.

	No. Genes	Best	Average	Worst
HHO-KNN	30	83.13%	60.64%	39.76%
	**29**	**91.57%**	56.43%	37.35%
	20	83.13%	53.41%	39.76%
	10	75.90%	47.83%	28.92%
	5	59.04%	42.41%	30.12%
HHO-SVM	30	90.36%	59.92%	33.73%
	**29**	**92.77%**	57.35%	40.96%
	20	83.13%	53.21%	34.94%
	10	78.31%	45.26%	21.69%
	5	69.88%	39.92%	24.10%

**Table 9:** Comparison between the proposed selection methods and previous methods in terms of the number of selected genes and accuracy.

Algorithms	Colon	Lung	Leukemia2	Leukemia3	Lymphoma	SRBCT
HHO-KNN	90.32%(16)	**100%(2)**	94.44%(11)	90.28%(25)	**100%(2)**	91.57%(29)
HHO-SVM	90.32%(10)	**100%(2)**	97.22%(11)	84.72%(25)	100%(3)	92.77%(29)
HS-GA [12]	95.9%(20)	-	97.5%(20)	-	-	-
FF-SVM [13]	92.7%(22)	**100%(2)**	99.5%(11)	-	92.6%(19)	97.5%(14)
GBC [14]	98.38%(20)	-	**100%(5)**	-	-	-
MIM-mMFA [15]	**100%(20)**	100%(20)	100%(6)	**100%(15)**	100%(4)	100%(23)
QMFOA [16]	100%(27)	100%(20)	100%(32)	100%(30)	-	100%(23)
BQPSO [17]	83.59%(46)	100%(46)	93.1%(48)	-	100%(49)	-
PCC-GA [18]	91.94%(29)	97.54%(42)	100%(35)	-	100%(39)	**100%(20)**

## Data Availability

Not applicable.
